# Complex scene green walnut target detection method based on improved YOLOv11n

**DOI:** 10.3389/fpls.2026.1971272

**Published:** 2026-10-14

**Authors:** Tingfeng Li, Xuanxi Yang, Ruoyu Li, Chen Xue, Zaiqing Chen, Lijun Yun

**Affiliations:** 1 School of Information, Yunnan Normal University, Kunming, China; 2 Engineering Research Center of Computer Vision and Intelligent Control Technology, Department of Education of Yunnan Province, Kunming, China; 3 Center for Planning and Policy Research, Yunnan Institute of Forest Inventory and Planning, Kunming, China

**Keywords:** complex scene, deep learning, green walnuts, object detection, YOLOv11n

## Abstract

To meet the efficiency and accuracy requirements of agricultural product inspection, deep learning-based object detection technology has been widely adopted. However, factors such as varying lighting conditions and foliage occlusion cause green walnuts in complex scenes to exhibit low texture contrast and a tendency to lose edge information. To address these issues, this paper proposes GPL-YOLO, an object detection algorithm for green walnuts based on an improved YOLOv11n architecture. First, the GhostModuleV2 module is introduced into the backbone network to enhance global modeling capabilities and enrich the detailed information of walnut targets within high-level feature maps. Second, a PPA module is integrated into the multi-scale feature fusion stage of the neck; this module effectively filters and enhances fused features across spatial and channel dimensions, thereby highlighting the target regions of green walnuts while suppressing background noise. Finally, a DW-LAE feature enhancement module is embedded in the downsampling fusion branch to adaptively preserve and enhance shallow-level detailed features during downsampling, thereby minimizing the loss of edge, texture, and shape information and improving the effectiveness of deep semantic features. Experimental results demonstrate that, compared to the baseline model, this algorithm achieves improvements of 8.6%, 5.7%, and 8.7% in Precision, Recall, and mAP50, respectively. The improved model outperforms the original YOLOv11n in detection accuracy and effectively mitigates missed detections and false positives in complex backgrounds, providing technical support for intelligent harvesting, yield estimation, and precision management in green walnut orchards.

## 1 Introduction

As a significant economic fruit crop, walnuts possess high nutritional and economic value, playing a pivotal role in my country’s fruit industry. Data regarding the quantity and spatial distribution of greened walnuts serves as a crucial basis for yield estimation, maturity assessment, intelligent harvesting, and precision orchard management. Traditional walnut yield statistics and fruit inspection mainly rely on manual labor. This method is easily affected by human experience and orchard environment, resulting in low efficiency and difficulty in achieving accurate fruit statistics. To improve orchard management efficiency and promote the intelligent development of the walnut industry, it is of practical significance to use image processing technology and target detection technology for automatic identification of green-skinned walnuts.

In recent years, driven by advancements in smart agriculture and agricultural automation technologies, deep learning-based object detection methods have been widely applied to tasks such as crop growth monitoring, fruit recognition, yield estimation, pest and disease detection, and visual positioning for harvesting robots. Research in fruit and vegetable detection has encompassed a variety of crops, including apples, citrus, tomatoes, strawberries, grapes, pears, peaches, mangoes, and walnuts. Automatic identification of fruit targets in field or orchard images can provide important data support for intelligent harvesting, precise pesticide application, yield prediction and refined orchard management (Rong et al., 2025). proposed a lightweight CT-MTD model to address the difficulties in estimating the maturity of tomatoes in greenhouse environments and the complexity of shading detection. This model uses the MobileVIT backbone for feature extraction and the CA module to enhance the dependency modeling of spatial and channel dimensions, thereby achieving effective discrimination of tomato maturity level and shading conditions (Kang et al., 2025). proposed a single-stage instance segmentation network to address the difficulties in identifying the maturity of broccoli and the inaccurate localization under shading conditions. This network can enhance the multimodal feature fusion capability and make the localization more accurate. Sun et al (Sun et al., 2025). proposed an Eggplant-DETR model to address the issues of significant scale differences in eggplant pest and disease targets in complex scenes and low recognition accuracy of occluded targets. This model integrates the CPSE module, CHSFPN feature pyramid, and wavelet transform multi-frequency attention mechanism, balancing detection accuracy and model lightweight.

Early fruit detection methods mostly relied on traditional image processing techniques, typically segmenting and identifying fruit regions using artificially designed features such as color, shape, and texture. For example, methods such as color space conversion (Horta-Velázquez et al., 2025), threshold segmentation (Chen et al., 2025), edge detection (Wang et al., 2026), and morphological processing (Yue et al., 2026) were used to extract target regions, which were then combined with classifiers to complete fruit identification. However, the natural orchard environment is highly complex and uncertain (Gong and Wu, 2025). The color of green walnuts is similar to the background of leaves, branches, etc., and is often accompanied by problems such as occlusion, overlap, uneven lighting, shadow interference, and changes in shooting angle. Traditional methods rely on artificial feature design, have limited feature expression capabilities, and are poorly adaptable to environmental changes (Jana and Parekh, 2017). In complex backgrounds, they are prone to target omissions, false detections, and inaccurate boundary positioning, making it difficult to meet the high-precision detection requirements in actual orchard scenarios.

With the advancement of deep neural networks, object detection has become a prominent topic in the field of computer vision. Object detection techniques are primarily categorized into two-stage and one-stage algorithms. Two-stage algorithms include Faster R-CNN (Ahmed and Das, 2025), Mask R-CNN (Zhang et al., 2024), and Cascade R-CNN (Chai et al., 2024). These algorithms effectively filter out background interference to achieve high-precision localization and recognition, resulting in lower rates of missed and false detections, particularly for small objects in complex scenes. However, two-stage algorithms involve two core processes, region proposal generation and precise object recognition, leading to slow inference speeds that fail to meet real-time requirements. Furthermore, they entail high computational overhead and deployment costs, while their complex model architectures result in lengthy development cycles. To address the complexity of two-stage algorithms, one-stage object detection algorithms were introduced. Examples include YOLO (Ali and Zhang, 2024), SSD (Zhao et al., 2024), and RetinaNet (Chen et al., 2024). These algorithms enable end-to-end real-time detection by eliminating the region proposal step; they perform object classification and bounding box regression in a single forward pass, making them widely applicable in scenarios such as edge computing and surveillance. In recent years, researchers have proposed various improved object detection algorithms to enhance both detection accuracy and real-time performance (Tian et al., 2025). proposed an improved YOLOv10s algorithm based on MFR (Multi-scale Feature Reconstruction) feature fusion; they designed an MFR mechanism to reconstruct features across different scales, effectively reducing missed and false detections and improving accuracy for aerial imagery. Ahmad et al., 2025) introduced DSFF-Net, a lightweight dynamic smooth feature fusion network; it utilizes DC modules to increase network depth while reducing computational costs and employs SA modules to suppress background noise, thereby mitigating interference when detecting small-to-medium objects in complex backgrounds or low-resolution images. To address challenges such as the high density and uneven distribution of small objects in aerial imagery (Hu et al., 2025), proposed MAfdet, a detection network architecture based on a fused multi-attention mechanism. By integrating a multi-attention focusing sub-network, a multi-scale Swin Transformer backbone, and a detection head, MAfdet achieves precise detection of small objects and significantly enhances the model’s feature extraction capabilities. To address the issues of low detection accuracy and excessive model parameters in infrared imagery (Cao et al., 2024), proposed YOLO-TSL, a lightweight algorithm based on an improved YOLOv8n; this model incorporates triple attention, integrates GSConv and GS modules, and features a cross-layer local network (VoV-GSCSP) to reduce computational complexity. Addressing the challenges posed by complex textural details and recognition difficulties (Zhang and Lei, 2024), introduced AFGN (Attention-Guided Fusion Network); this algorithm incorporates an Attention Feature-Guided Branch (AFGB) into the training phase of a CNN-based end-to-end detection network and utilizes a novel background-blurring mask embedded within the AFGB to focus the model on objects amidst complex backgrounds. To meet the demands of complex scene modeling, high precision, multi-task fusion, and open world scenarios, the Google team proposed the Transformer model (Vaswani et al., 2017), built entirely on the self-attention mechanism for sequence modeling, which achieves end-to-end set prediction, thereby simplifying the workflow while enhancing recognition accuracy. Subsequently, the Facebook team introduced the DETR architecture (Carion et al., 2020) based on the Transformer structure; this method eliminates traditional components such as region proposals and non-maximum suppression, delivering superior performance in detecting large objects. However, due to the high computational cost of DETR, manifested in slow convergence, training, and inference speeds that fail to meet real-time requirements (Zhao et al., 2024). proposed RT-DETR (Real-Time Detection Transformer), which handles multi-scale features by decoupling intra-scale interactions from cross-scale fusion, thereby increasing speed while maintaining accuracy.

Currently, YOLO-series algorithms are widely applied in research on defect detection and grading for agricultural products such as tomatoes and chili peppers. To address issues like blurred boundaries, missed detections, and occlusion of tomato fruits under complex lighting conditions (Li et al., 2026), proposed a lightweight YOLO-MSRF framework; this framework incorporates MS-CPAM and features a dual-branch multi-modal backbone alongside a multi-scale fusion and semantic refinement network (MSF-SRNet), thereby enhancing segmentation accuracy and overall performance. Addressing challenges related to occlusion and fruit overlapping (Zhao et al., 2026), proposed a lightweight RED-YOLO algorithm that integrates the CBAM attention mechanism with GSConv and VoV-GSCSP modules; this approach achieves model lightweighting while improving recognition accuracy for red chili pepper clusters. To tackle high error rates in local defect detection (Xue et al., 2026), proposed an enhanced DBH-YOLO algorithm; by replacing the original model’s C2f module with DGST, incorporating an LSKA-based spatial pyramid structure, and adding a DyHead dynamic detection head, the algorithm improved surface defect detection capabilities for Lonicera caerulea (blue honeysuckle) berries. Finally, to address the issue of missing potted vegetable seedlings (Yu et al., 2026), proposed the SD-MSL-YOLO algorithm; this method utilizes a GLSA-BiFPN structure to enhance representation capabilities and incorporates TADDH to mitigate environmental noise, thereby improving both seedling detection and the localization of missing seedlings. To enhance walnut recognition under complex lighting (Wu et al., 2024), redesigned the feature extraction and feature fusion networks of the YOLOv8 model, improving detection accuracy while maintaining a lightweight architecture. To further improve detection capabilities for occluded walnuts (Wang et al., 2025a), categorized walnuts into occluded and non-occluded groups and proposed the OW-YOLO method; this approach involved reconstructing the YOLOv8 backbone and redesigning the detection head to boost accuracy while reducing model complexity (Wang et al., 2025b). proposed the RSWD-YOLO method, which reconstructs the feature fusion network to enhance detection accuracy and enable deployment on small-scale edge devices. Liu et al (Liu, 2024). incorporated a multi-scale feature dynamic convolution kernel into the YOLOv8 model to improve the recognition accuracy of greened walnuts.

In summary, while existing target detection methods and agricultural fruit and vegetable detection methods have made some progress, the following problems still exist in the detection scenario of green walnuts in natural orchards. First, complex backgrounds of branches and leaves and changes in lighting can easily interfere with the model’s judgment, making it difficult to distinguish walnuts from the background. Second, green walnuts are small in scale and often overlap or occlude, resulting in insufficient feature representation of small and occluded targets by the model. Third, the multi-scale feature fusion and downsampling processes lack sufficient preservation and filtering of effective detailed information, easily leading to the loss of target texture, edge, and shape features.

To address the aforementioned issues, this paper proposes an improved model, GPL-YOLO, for green walnut detection in complex scenes based on YOLOv11n. This model mainly incorporates improvements in three aspects: the backbone network, neck multi-scale feature fusion, and downsampling structure. The main contributions of this paper are as follows:

1. A green walnut detection model, GPL-YOLO, tailored for complex scenes, is proposed. This model specifically addresses issues such as the small size of green walnut targets and complex backgrounds by making targeted improvements to the network structure of YOLOv11n, aiming to enhance the localization and recognition capabilities of green walnut targets in complex scenes.
2. Introduce the GhostNetV2 module into the backbone network, leveraging its feature generation mechanism and long-range dependency modeling capabilities to enhance the model’s ability to extract edge, texture, shape, and contextual details of green walnuts, thereby improving the distinction between the target and complex backgrounds.
3. Introduce the PPA module in the multi-scale feature fusion stage of the neck, effectively screen the fused features in spatial and channel dimensions, highlight target regions and key features, suppress complex background noise, and improve the model’s ability to express features of small and occluded targets.
4. Replace the standard downsampling convolution in the bottom-up path of the neck with the DW-LAE module, so that the model can effectively preserve and enhance shallow-layer detail information during the downsampling process, reduce the loss of fruit edges, textures, and shape features, and thereby enhance the representation ability of green walnut targets in deep semantic features.
5. Through comparative and ablation experiments, the effectiveness of the proposed model and its improved modules is verified. The experimental results show that GPL-YOLO exhibits good performance in detecting green walnuts in complex scenes and possesses potential for application in automatic identification of green walnuts, yield prediction, and intelligent management of orchards.

## 2 Materials and methods

### 2.1 Data acquisition

To construct an image dataset suitable for aerial target detection of green-skinned walnuts, this study conducted drone image acquisition in Yangbi County, Dali Bai Autonomous Prefecture, Yunnan Province, China, from July 18 to September 14, 2024. Eight walnut-growing plots were selected as sampling areas. Each plot exhibited different canopy morphology, planting density, and fruit distribution characteristics, which can effectively recreate the true growth state of green-skinned walnuts in a natural orchard setting.

To account for the effects of varying natural light on the color, texture, and edge characteristics of greened walnuts, image acquisition was scheduled between 09:00 and 19:00 daily, capturing samples under diverse solar elevation angles and light intensities. A DJI Matrice 300 RTK drone, equipped with a Zenmuse P1 camera and a 35 mm F2.8 lens, was used for data collection. Ultimately, a total of 9,399 raw aerial images were obtained across the eight study plots.

Given that high flight-path overlap results in redundant areas across adjacent images, and can even lead to the same walnut tree being captured multiple times, we manually screened the raw aerial imagery during the data preparation stage. We examined the images from each plot individually, discarding samples characterized by highly similar fields of view, excessive overlap, or poor image quality; this process aimed to ensure that the retained images exhibited low overlap and high scene diversity, thereby enhancing the effectiveness of the dataset.

### 2.2 Data preprocessing

The original aerial images have a resolution of 8192×5460 pixels, which would incur high computational costs if used directly for model training. Furthermore, scaling them directly to the network input size could result in loss of detail in small green walnut targets. Therefore, this paper employs a sliding window approach to slice the selected images, with a cropping window size of 1024×1024 pixels and a sliding step of 512 pixels. After image selection and slicing, a final dataset of 30,240 aerial images for green walnut detection is constructed.

To analyze the impact of complex natural environments on detecting green walnuts, this article labels targets based on two aspects: lighting conditions and occlusion. Lighting can change the brightness, color, texture of the fruit area and its contrast with the background, while occlusion affects the completeness of the fruit outline and visible area; both are key factors that make detection more difficult. Therefore, green walnuts are divided into four categories: front-lit and unoccluded (A1), back-lit and unoccluded (A2), front-lit and occluded (B1), and back-lit and occluded (B2). As shown in **Figure 1**, the blue box represents front-lit unoccluded (A1), the orange box is back-lit unoccluded (A2), the white box shows front-lit occluded (B1), and the green box indicates back-lit occluded (B2). This classification helps track sample distribution under various complex conditions and further assess the model’s performance under changing light and occlusions.

**Figure 1:**
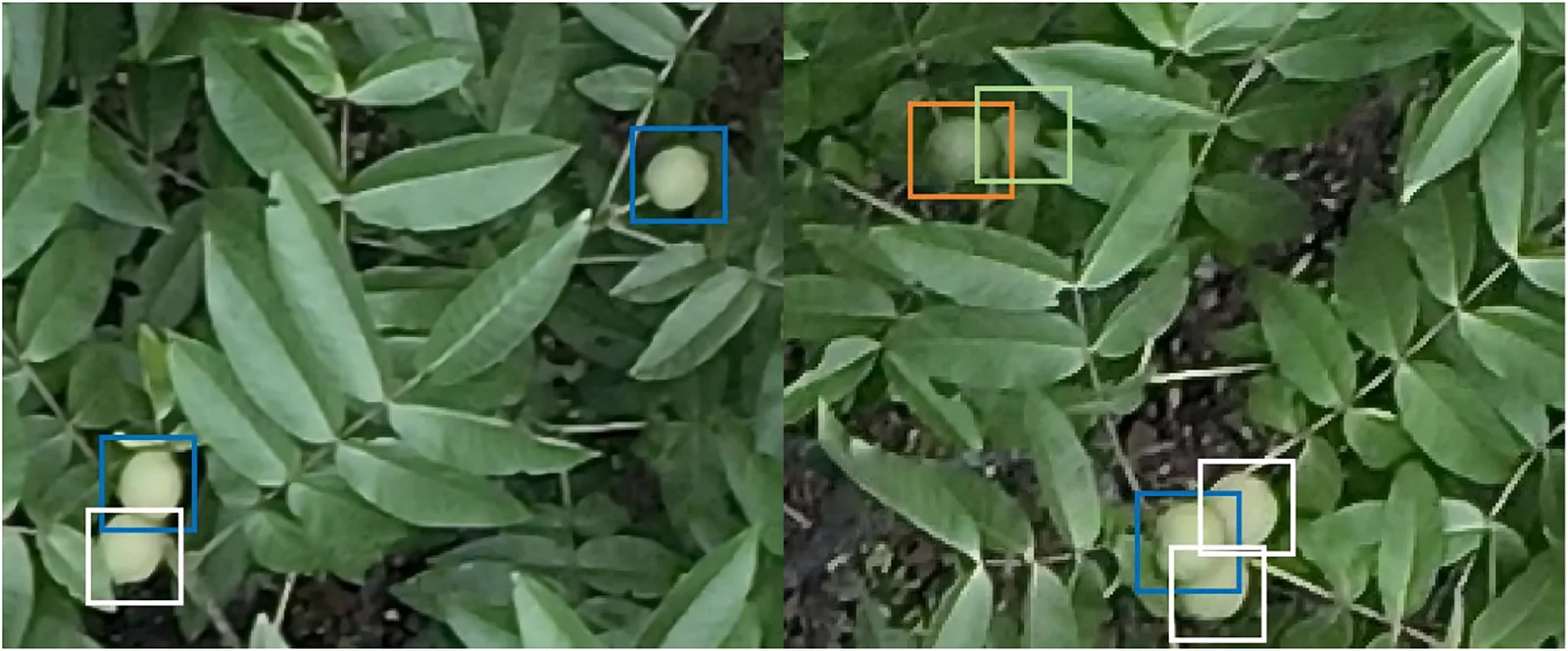
Walnut category labels.

The LabelImg annotation tool was used to annotate the dataset, resulting in a total of 24,673 labels. Following the partitioning conventions of mainstream object detection models, the dataset was divided into training, validation, and test sets at a ratio of 7:2:1, comprising 21,167, 6,048, and 3,025 images, respectively. Based on the COCO dataset definitions for small, medium, and large objects, the walnut targets were categorized accordingly. **Table 1** presents detailed statistics for the dataset, while **Figure 2** illustrates the distribution of large, medium, and small targets across the sets. Within the dataset, the counts of large targets in the training, validation, and test sets were 526, 175, and 74, respectively; these represent a small proportion of the total, with the majority consisting of small and medium-sized targets—a distribution consistent with the morphological characteristics of walnut fruit as viewed from a drone.

**Table 1:** Statistics of dataset information.

Datasets	Split	Image quantity	BBox quantity
WalnutData	Train	21,167	495,812
	Val	6,048	139,255
	Test	3,025	71,141

**Figure 2:**
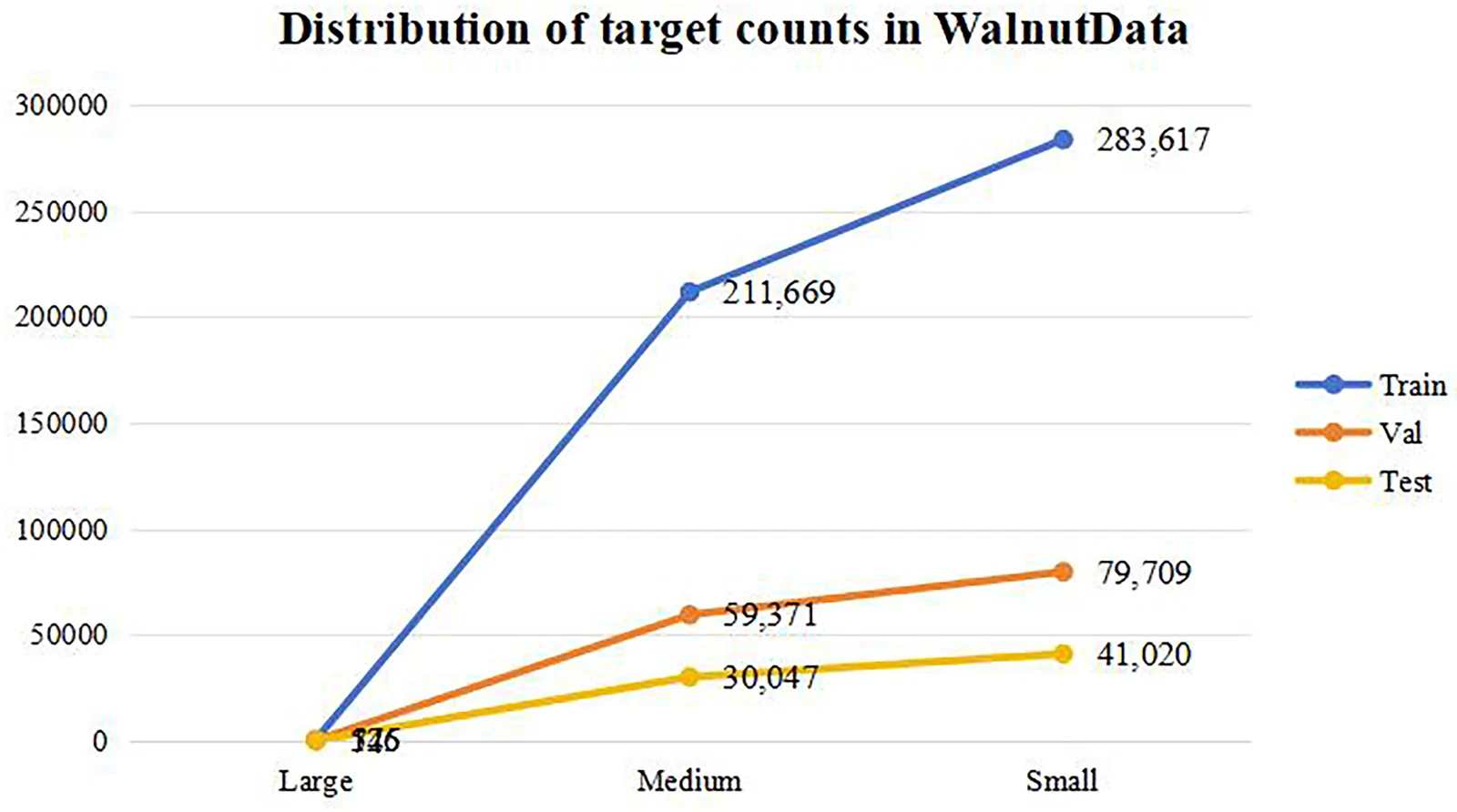
Distribution of the number of large, medium, and small objects in WalnutData.

### 2.3 Overall framework

The YOLOv11n network architecture primarily consists of three components: the backbone, the neck, and the detection head. The backbone extracts multi-scale feature information from input images; the neck enhances information interaction across different scales through feature fusion; and the detection head performs object classification and bounding-box regression based on the fused feature maps to achieve multi-scale detection. To address challenges such as lighting variations, occlusion, and background interference encountered when detecting walnuts in natural environments, this paper utilizes YOLOv11n as the baseline model. YOLOv11n was chosen as the base detector because it provides a favorable trade-off between detection performance and computational efficiency. Compared to the larger YOLOv11 variant, YOLOv11n has fewer parameters and is more suitable for deployment on resource constrained devices. It can be used to identify target objects in complex backgrounds and constantly changing lighting conditions. Therefore, YOLOv11n is considered a suitable model for this study. GhostNetV2 is introduced to strengthen the backbone’s feature extraction capabilities, while PPA and DW-LAE modules are incorporated to improve the model’s perception of key regions and local details. Ultimately, an improved walnut detection model, named GPL-YOLO, is constructed. **Figure 3** illustrates the overall architecture of the model.

**Figure 3:**
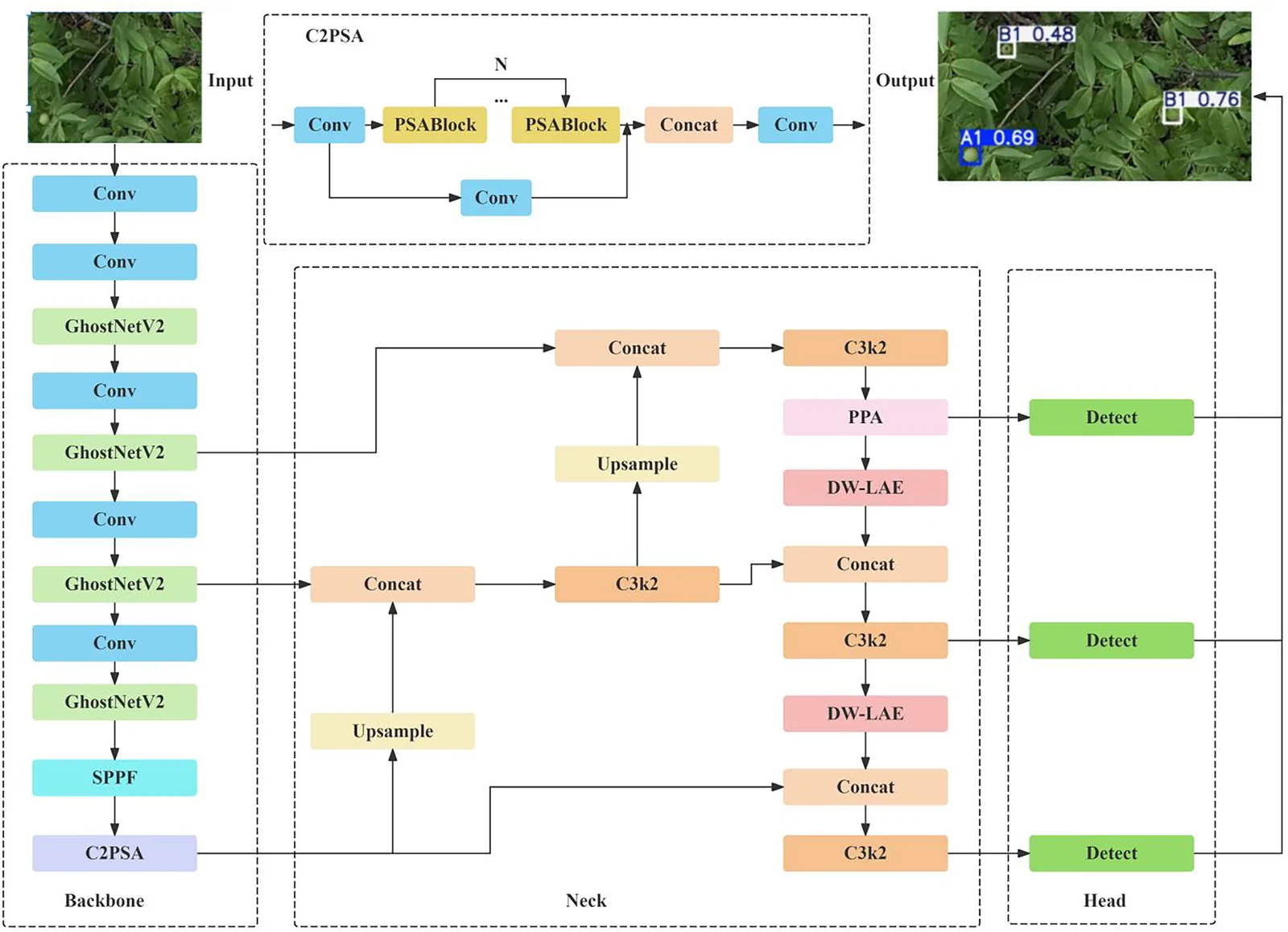
Overall architecture of GPL-YOLO.

GPL-YOLO incorporates three improvements over the original YOLOv11n model. First, we enhance the feature extraction network to boost the backbone’s ability to extract features from small objects at shallower layers. Second, we optimize the neck’s feature fusion structure by introducing a PPA attention module after the shallow P3 branch; this reinforces fine-grained shallow features, suppresses background noise, and improves small-object detection performance. Finally, we embed a DW-LAE feature enhancement module into the downsampling fusion branch to optimize cross-level feature fusion efficiency, thereby mitigating the loss of feature information inherent in the original PANet fusion process.

### 2.4 GhostNetV2 module

From the perspective of a drone, green walnut targets are relatively small, mostly medium to small in size. The YOLOv11 backbone network, during feature extraction, undergoes multiple convolutions and downsampling, which can weaken detailed information such as walnut edges, textures, and contours. The C3k2 module primarily relies on local convolutions for feature extraction, lacking sufficient modeling capabilities for long-range spatial dependencies and global contextual information. This can lead to inadequate expression of green walnut target features. Therefore, it is necessary to introduce a feature extraction module with local detail enhancement and global context modeling capabilities to improve the model’s ability to extract features from green walnut targets.

To address the aforementioned issues, this study replaces the C3K2 module of the backbone network with GhostNetV2. **Figure 4** illustrates the architecture of GhostNetV2. The GhostNetV2 (Tang et al., 2022) module consists of two parallel branches. Since the Ghost module interacts with only a subset of pixels, its capability to capture spatial information is limited. Therefore, DFC attention is employed to re-establish long-range spatial dependencies and enhance the output features Y of the Ghost module. The input features $X\in R^{H\times W\times C}$ are fed into the two branches separately: one branch utilizes the Ghost module to produce output features Y, while the other employs the DFC module to generate an attention map A. A 1×1 convolution is used to transform the input features X into the input Z for the DFC module. The final output $O\in R^{H\times W\times C}$ is the product of the two branches, as shown in Equation 1.

**Figure 4:**
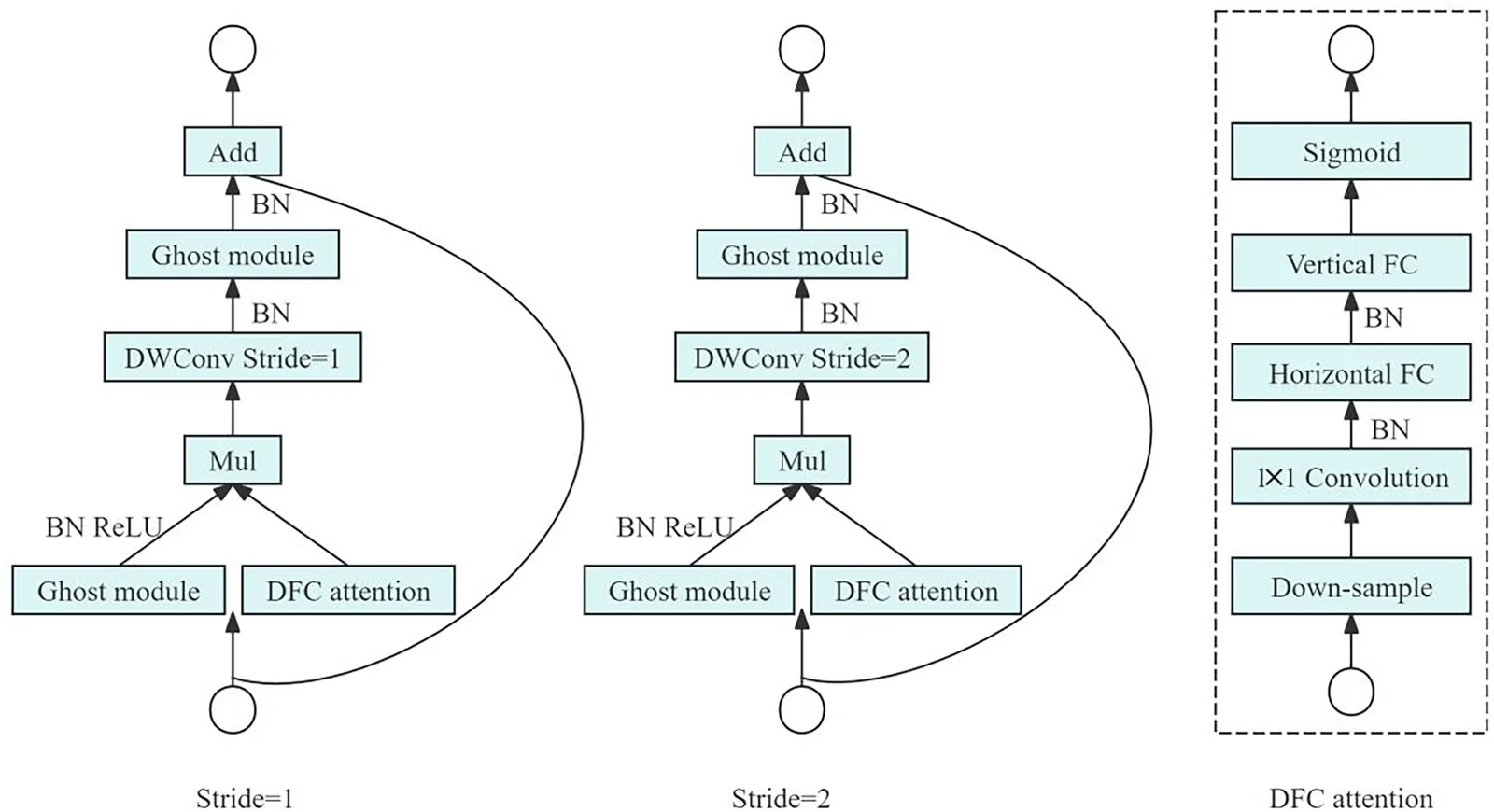
GhostNetV2 architecture diagram.

(1) O=Sigmoid(A)⨀V(X)

where ⊙ denotes element-wise multiplication, V(X) denotes the feature representation obtained by applying the transformation function to the input feature X, and the Sigmoid function serves as a scaling function that normalizes the attention map A to the range (0, 1). The input features incorporate both local information extracted by the Ghost module and global spatial dependency information extracted by the DFC, thereby enhancing the model’s feature representation and spatial perception capabilities.

In this study, GhostNetV2 replaces the C3k2 module in the YOLOv11n backbone network to enhance its feature extraction capabilities. Compared to C3k2, GhostNetV2 pays more attention to global information. GhostNetV2 extracts local details such as edges, textures, and contours of green walnuts through the Ghost branch and models long-distance spatial dependencies using the DFC attention branch, enhancing the network’s ability to perceive global contextual information. Therefore, this module can better distinguish green walnuts from branches and leaves in complex backgrounds. Simultaneously, GhostNetV2 provides feature maps with richer details and contextual information for subsequent feature fusion. Therefore, introducing GhostNetV2 into the green walnut detection network helps improve the model’s detection accuracy in complex natural backgrounds and reduces the problems of missed and false detections of small targets.

### 2.5 Parallelized patch-aware attention module

Although replacing the C3k2 module in the YOLOv11n backbone with GhostNetV2 enhances the network’s ability to extract edges, textures, contours, and contextual information regarding green walnuts, thereby providing richer feature maps for subsequent fusion, challenges remain during the multi-scale feature fusion process. Because feature maps at different levels (such as P3, P4, and P5) vary in size, scale alignment via upsampling or downsampling is required prior to fusion; this process often leads to the degradation of edge and texture details, resulting in blurred feature maps after upsampling. Regarding green walnut targets viewed from a UAV, high-level features contain strong semantic information but lack detail, whereas low-level features possess rich spatial and textural information but are susceptible to interference from complex backgrounds like foliage and shadows. Consequently, the fusion of deep semantic features and shallow spatial features can result in spatial misalignment and feature redundancy, leading to the inclusion of significant background noise in the fused feature maps.

To address the aforementioned issues, this paper introduces a PPA module during the feature fusion stage to further filter, enhance, and calibrate the fused features. **Figure 5** illustrates the structure of the PPA module. The PPA (Xu et al., 2024) employs a multi-branch feature extraction strategy consisting of three parallel branches: local, global, and convolutional. An input feature map $F\in R^{H^{\prime }\times W^{\prime }\times C}$ undergoes pointwise convolution for dimensionality alignment to yield $F^{\prime }\in R^{H^{\prime }\times W^{\prime }\times C^{\prime }}$. It then passes through the three branches to compute $F_{local}\in R^{H^{\prime }\times W^{\prime }\times C^{\prime }}$, $F_{global}\in R^{H^{\prime }\times W^{\prime }\times C^{\prime }}$ and $F_{conv}\in R^{H^{\prime }\times W^{\prime }\times C^{\prime }}$. Finally, the outputs from these three branches are summed element-wise to produce the ultimate fused feature map $F_{fusion}\in R^{H^{\prime }\times W^{\prime }\times C^{\prime }}$.

**Figure 5:**
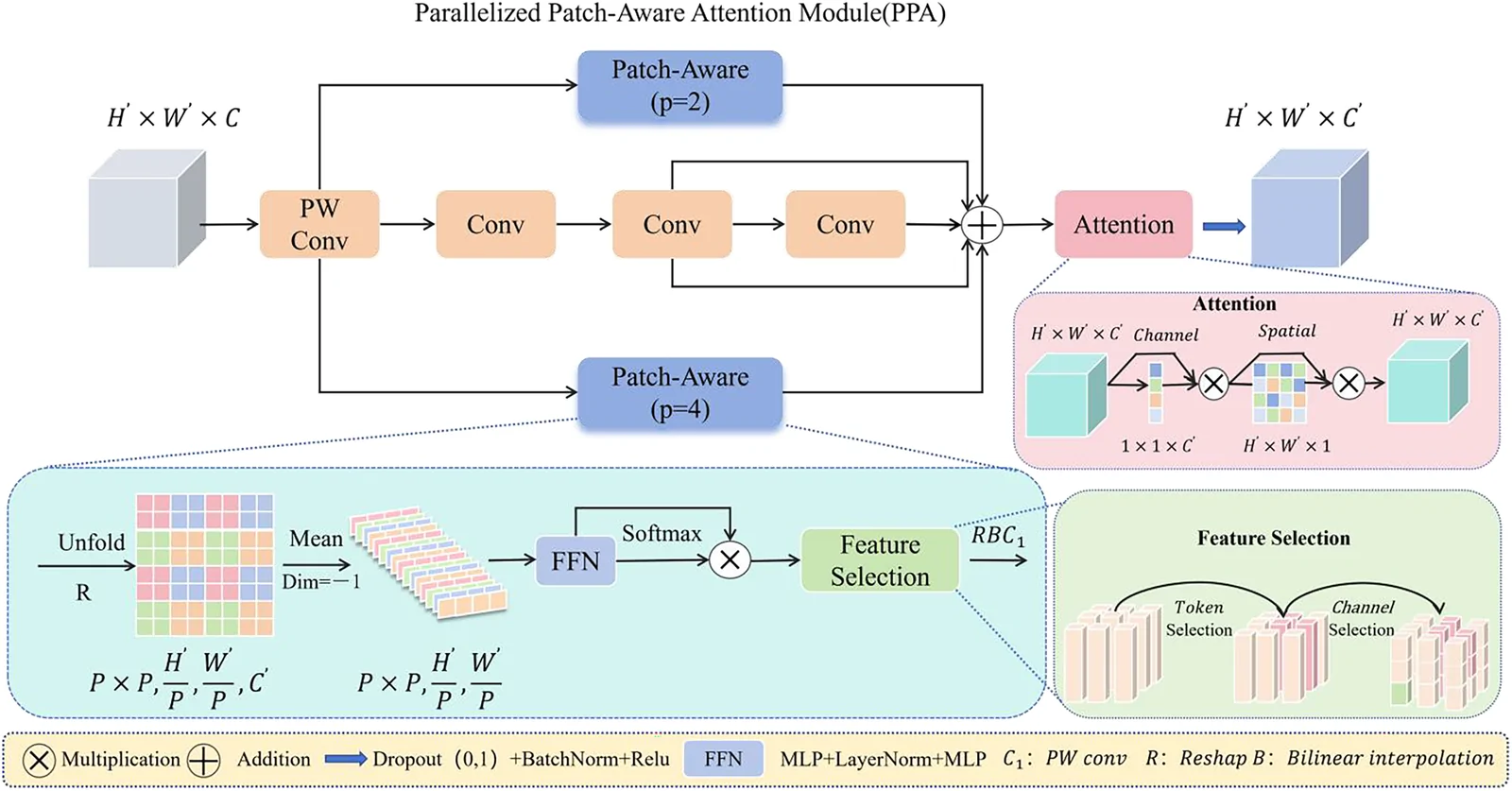
PPA structure diagram.

Local and global branches are distinguished by a patch size parameter p, which is implemented through the grouping and displacement of non-overlapping patches along the spatial dimensions. First, the feature map $F^{\prime }\in R^{H^{\prime }\times W^{\prime }\times C^{\prime }}$ is partitioned into non-overlapping patches ( $P\times P,\frac{H^{\prime }}{P},\frac{W^{\prime }}{p},C^{\prime }$) via unfolding and reshaping operations. Subsequently, the mean is calculated along the channel dimension to yield a representation of shape ( $P\times P,\frac{H^{\prime }}{P},\frac{W^{\prime }}{p}$). A feed-forward network (FFN) performs a linear transformation, followed by an activation function to derive a spatial probability distribution for the linearly computed features; these weights are then multiplied by the original patch features to produce weighted features. Token and channel selection are applied to the weighted features to retain target-relevant tokens. Finally, reshaping, interpolation, and convolution operations are used to restore the original dimensions, yielding $F_{local}\in R^{H^{\prime }\times W^{\prime }\times C^{\prime }}$ and $F_{global}\in R^{H^{\prime }\times W^{\prime }\times C^{\prime }}$. The intermediate convolutional branch employs a sequence of three convolutional layers; the resulting three feature maps are summed element-wise to produce the branch output $F_{conv}\in R^{H^{\prime }\times W^{\prime }\times C^{\prime }}$. Ultimately, building upon the multi-branch feature extraction, an attention mechanism is utilized for adaptive feature enhancement. The attention module incorporates efficient channel attention and spatial attention. The fused feature map $F_{fusion}\in R^{H^{\prime }\times W^{\prime }\times C^{\prime }}$ is processed by the channel attention map $M_{c}\in R^{1\times 1\times C^{\prime }}$ and the spatial attention map $M_{s}\in R^{H^{\prime }\times W^{\prime }\times 1}$, followed by a Dropout operation, to output the final enhanced feature $F^{\prime \prime }\in R^{H^{\prime }\times W^{\prime }\times C^{\prime }}$, as detailed in (Equation 2) and (Equation 3).

(2) $$F_{c}=M_{c}\left(F_{fusion}\right)⊗F_{fusion},\;F_{s}=M_{s}\left(F_{c}\right)⊗F_{c},$$

(3) $$F^{\prime \prime }=\delta \left(ℬ\left(dropout\left(F_{s}\right)\right)\right),$$

where ⊗ denotes element-wise multiplication; $F_{c}\in R^{H\times W\times C^{\prime }}$ and $F_{s}\in R^{H\times W\times C^{\prime }}$ represent the features after channel and spatial selection, respectively; δ(·) and ℬ(·) denote the Rectified Linear Unit (ReLU) and Batch Normalization (BN), respectively; and $F^{\prime \prime }\in R^{H^{\prime }\times W^{\prime }\times C^{\prime }}$ is the final output of the PPA.

The PPA module leverages the rich features extracted by the GhostNetV2 backbone and employs a multi-branch structure to capture local details and global semantic information at varying granularities, thereby compensating for the loss of fine-grained features, such as edges and textures, that typically occurs during scale transformations. Specifically, the local branch reinforces the edge, texture, and shape characteristics of small targets, enhancing localization accuracy; the global branch establishes connections between local details and holistic semantics, improving the model’s ability to distinguish green walnuts from visually similar background elements like leaves and branches; and the attention mechanism applies weights to the fused features, prioritizing walnut-related regions while suppressing background noise and redundant information. By incorporating the PPA, the model more effectively preserves and enhances target features during the fusion stage, providing the detection head with superior fused feature maps; this reduces missed detections and false positives, ultimately improving detection accuracy in complex orchard environments.

### 2.6 DW-LAE module

In the bottom-up feature fusion path of the neck, standard convolutions are typically employed to downsample shallow-layer features, aligning their spatial dimensions with those of deeper-layer features prior to concatenation and fusion. While this approach achieves scale alignment, the use of standard downsampling convolutions often leads to the loss of fine-grained details, such as edges, textures, contours, and shapes, during the reduction of feature map dimensions. In the context of detecting green walnuts from a UAV perspective, standard convolutions lack the ability to adaptively select relevant regions and critical channels; consequently, background noise and redundant features may propagate to deeper layers, interfering with the semantic features at the P5 level and increasing the risk of the model misclassifying background elements, such as branches, foliage, or shadows, as walnuts.

To mitigate the loss of detail and the propagation of background noise associated with standard downsampling convolutions, this paper replaces the standard convolution in the bottom-up path of the Neck with the DW-LAE module. **Figure 6** illustrates the structure of the DW-LAE module. The DW-LAE module consists of two parallel branches, lightweight extraction and adaptive extraction, designed to simultaneously capture spatial and channel feature dependencies in walnut images characterized by complex backgrounds, indistinct textural features, and significant variations in walnut scale. These branches process features independently; the resulting feature weights are multiplied together and then fused with the original features to produce enhanced downsampled features. In the lightweight extraction branch, the input feature map $F\in R^{h\times w\times c}$ first undergoes grouped convolution with Groups=c/16. Subsequently, the output features are rearranged and mapped to spatial weights, suppressing background noise and focusing on the walnut target regions. In the adaptive extraction branch, the input feature map first undergoes depthwise convolution, followed by 1×1 convolution to extract inter-channel dependencies; after dimension rearrangement and mapping, a normalization operation generates channel attention weights, which attenuate redundant background channels and enhance feature representation in key channels. Finally, the spatial weights from the lightweight branch and the channel weights from the adaptive branch are multiplied element-wise and fused with the original walnut features to output the feature map $F^{\prime }\in R^{h/2\times w/2\times c}$.

**Figure 6:**
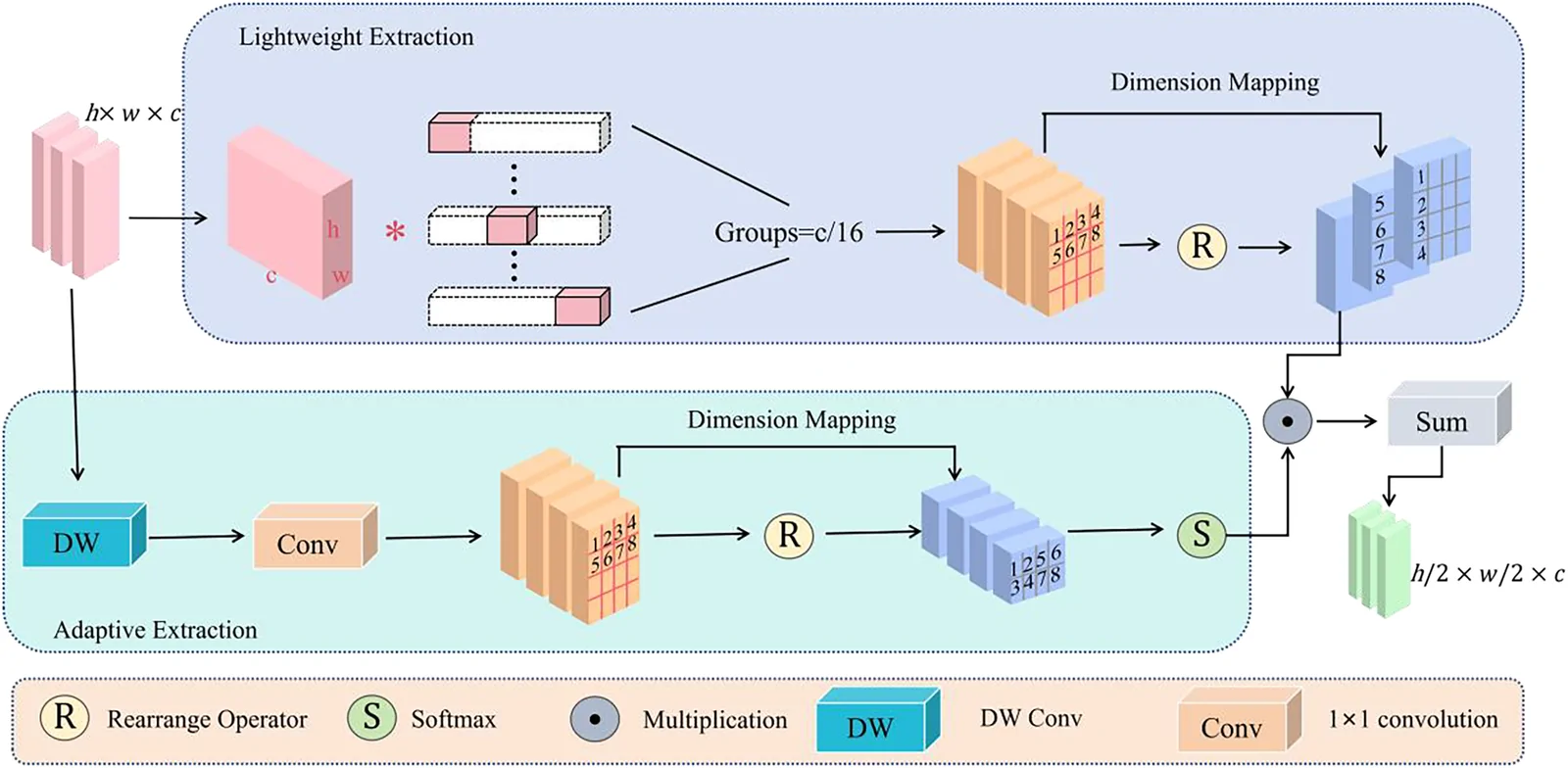
Structure diagram of DW-LAE.

Compared with the original LAE (Yu et al., 2024) which uses AvgPool for feature aggregation, DW-LAE uses 3×3 DW Conv to replace average pooling, so that the downsampling process is no longer just smoothing and compressing local areas, but can learn the local texture, edge and shape features of the walnut target, and adaptively adjust the feature response according to different scale targets. At the same time, DW Conv has the characteristics of few parameters and low computational cost, which can improve the detail preservation ability while maintaining the model’s lightweightness. By replacing ordinary downsampling convolution with DW-LAE, the model can more effectively transfer the shallow detail information enhanced by PPA to the deep features, improve the deep network’s perception ability of small green walnut targets, and thus reduce the false detection and false detection rates in complex orchard scenarios. **Table 2** lists the walnut recognition performance of the original LAE and DW-LAE.

**Table 2:** Experimental results of the LAE module.A1, front-lit and unoccluded;. A2, back-lit and unoccluded; B1, front-lit and occluded; B2, back-lit and occluded.

Method	A1	A2	B1	B2	mAP50(%)	mAP50:95(%)	Parameters(M)	GFLOPs
Baseline(YOLOv11n)	84.0	79.7	68.5	63.7	74.0	48.0	2.6	6.3
Baseline+LAE1	83.6	79.4	68.9	64.4	74.1	48.1	2.3	6.4
Baseline+LAE2	83.9	79.6	68.7	64.0	74.0	48.2	2.5	6.3
Baseline+LAE3	83.9	79.8	68.9	64.7	74.3	48.7	2.5	6.3
Baseline+LAE4	84.4	80.2	69.0	64.7	74.6	48.7	2.5	6.3

## 3 Results and discussion

### 3.1 Experimental setup

We used YOLOv11n as the baseline model and the WalnutData (Wu et al., 2025) dataset; the evaluation metrics included precision (P), recall (R), mean average precision (mAP), and F1-score. All models were trained using an NVIDIA GeForce RTX 5060Ti (16 GB) GPU, Python 3.10.18, PyTorch 2.7.0, and CUDA 12.8.

The baseline model used is YOLOv11n; to ensure consistency across variables, the training duration was set to 300 epochs for all experiments. **Table 3** lists the key parameters employed during the experiments.

**Table 3:** Experimental parameter settings.

Epoch	Batch	Workers	Initial learning rate	Optimizer	Momentum	Weight deacy
300	16	4	0.01	SGD	0.937	0.0005

### 3.2 Ablation study

We conducted ablation studies on the proposed modules to verify their individual effectiveness and their interrelationships. As shown in **Table 4**, we performed experiments to validate the efficacy of each model component, specifically the GhostNetV2, PPA, and LAE modules. The results indicate that incorporating GhostNetV2 improved detection precision by 8.6%, recall by 5.1%, mAP50 by 8.4%, mAP50:95 by 7.9%, and F1-score by 7%. These findings demonstrate that the GhostNetV2 module effectively enhances walnut detection capabilities, mitigates issues arising from the similarity between walnut appearance (color and texture) and the background, and reduces missed detections caused by foliage occlusion. Upon introducing the PPA module, precision increased by 0.3%, recall by 0.3%, mAP50 by 0.4%, mAP50:95 by 1.1%, and F1-score by 1%; this shows that PPA alleviates poor localization of small targets caused by multiple downsampling operations. The introduction of the DW-LAE module resulted in a 0.7% increase in precision, 0.2% in recall, 0.6% in mAP50, 0.7% in mAP50:95, and 1% in F1-score-score, demonstrating its ability to enhance multi-scale feature fusion and improve walnut recognition performance. Finally, by integrating all three modules (GhostNetV2, PPA, and DW-LAE), the model achieved improvements of 8.6% in precision, 5.7% in recall, 8.7% in mAP50, 8.5% in mAP50:95, and 8% in F1-score. These results confirm that the proposed method is effective: GhostNetV2 provides feature maps rich in detail and semantic information for fusion; PPA extracts information at varying granularities and mitigates the loss of edge texture details caused by upsampling at the P4 layer; and DW-LAE adapts to walnut features across different scales, thereby strengthening feature fusion capabilities. The combination of the three modules effectively enhances the detection capability for walnut targets against complex backgrounds, effectively reduces missed detections and false positives, and improves localization accuracy.

**Table 4:** Ablation study.

GhostNetV2	PPA	DW-LAE	P(%)	R(%)	mAP50(%)	mAP50:95(%)	F1-score
*×*	*×*	*×*	65.5	71.8	74.0	48.0	68.0
✓	*×*	*×*	74.1	76.9	82.4	55.9	75.0
*×*	✓	*×*	65.8	72.1	74.4	49.1	69.0
*×*	*×*	✓	66.2	72.0	74.6	48.7	69.0
✓	✓	*×*	74.1	77.1	82.7	56.5	75.0
✓	*×*	✓	73.3	76.9	82.1	55.7	75.0
*×*	✓	✓	65.7	71.9	73.8	48.5	69.0
✓	✓	✓	74.1	77.5	82.7	56.5	76.0

### 3.3 Comparative experiments

To verify the effectiveness of the GPL-YOLO algorithm proposed in this paper in detecting green walnuts in complex natural orchard environments, YOLOv11n is selected as the baseline model and compared with representative object detection algorithms in recent years. The comparison models include YOLOv3-tiny, YOLOv5n, YOLOv6n, YOLOv7, YOLOv8n, YOLOv9t, YOLOv10n, YOLOv12n, YOLOv26n, RT-DETR, Faster R-CNN, FCOS, and RetinaNet. The aforementioned models cover one-stage detectors, two-stage detectors, and Transformers-based detectors, enabling the evaluation of GPL-YOLO’s detection performance from the perspective of different network structures. **Table 5** lists the AP, mAP50, F1-score, and other evaluation metrics for each model.

**Table 5:** Comparison of experimental results. A1, front-lit and unoccluded;. A2, back-lit and unoccluded; B1, front-lit and occluded; B2, back-lit and occluded.

Name	A1	A2	B1	B2	mAP50 (%)	F1-score	Parameters(M)	GFLOPs
YOLOv3-tiny	73.3	70.9	64.0	53.1	65.3	61.0	8.7	12.9
YOLOv5n	80.2	77.5	68.5	60.2	71.6	66.0	1.8	4.1
YOLOv5s	88.0	86.9	82.5	74.9	83.1	76.0	7.0	15.8
YOLOv6n	81.4	75.9	63.2	58.0	69.6	65.0	4.2	11.7
YOLOv7	80.8	75.9	64.5	56.9	69.5	65.0	6.0	13.2
YOLOv8n	84.2	79.6	68.4	63.7	74.0	69.0	3.0	8.1
YOLOv9t	83.8	79.4	67.6	62.1	73.2	68.0	2.0	7.6
YOLOv10n	84.1	79.8	69.2	64.7	74.5	69.0	2.7	8.2
YOLOv11n	84.0	79.7	68.5	63.7	74.0	68.0	2.6	6.3
YOLOv12n	83.8	79.5	69.5	65.4	74.6	69.0	2.6	6.3
YOLOv26n	84.0	79.5	68.7	64.5	74.2	69.0	2.4	2.9
RT-DETR	75.9	72.5	61.7	55.8	66.5	70.0	42.8	130.5
Faster R-CNN	93.2	86.4	80.3	86.6	86.6	85.3	41.4	76.8
FCOS	91.5	89.9	84.9	84.5	87.7	84.3	32.1	80.3
RetinaNet	82.4	76.6	68.3	70.8	74.5	72.1	32.3	81.7
Ours(GPL-YOLO)	89.3	86.3	78.5	76.7	82.7	76.0	5.2	12.6

Compared to the YOLOv11n baseline model, The mAP50 of GPL-YOLO has increased by 8.7% and an 8% increase in F1-score, with detection accuracy improving for both unoccluded and occluded walnuts. These experimental results demonstrate that the improved algorithm effectively enhances the distinct representation of walnut targets against the background and improves the model’s ability to differentiate walnuts from background elements such as branches and leaves. This method effectively suppresses the misclassification of background elements as walnut targets while incorporating fine-grained features such as walnut texture and edges into a multi-scale feature pyramid, thereby improving detection accuracy.

The experimental results indicate that model networks such as YOLOv3-tiny, YOLOv5n, YOLOv6n, YOLOv7, YOLOv8n, YOLOv9t, YOLOv10n, YOLOv12n, YOLOv26n, RT-DETR, and RetinaNet generally suffer from deficiencies in shallow-layer detail feature extraction and weak global long-distance dependency modeling capabilities. Faced with the impacts of uneven illumination and foliage occlusion, these models are prone to issues such as missed and false target detections, with mAP50 values consistently lower than those achieved by the improved algorithm presented in this paper. Although Faster R-CNN and FCOS exhibit higher detection accuracy than GPL-YOLO, these two models also have relatively larger parameter counts and computational demands, along with more complex model structures and computational processes. This poses certain limitations in real-time detection and edge device deployment within the actual orchard environment. In contrast, although GPL-YOLO does not achieve the highest detection accuracy among all comparative models, it effectively controls the model’s parameter count and computational overhead while maintaining high detection accuracy. It strikes a good balance between detection accuracy, computational efficiency, and adaptability to complex scenarios.

Furthermore, test results under different lighting and occlusion conditions reveal that the improved algorithm outperforms all comparable YOLO models in both frontal and backlighting environments, as well as in both unoccluded and occluded walnut detection scenarios. In conclusion, the proposed algorithm better adapts to real-world detection environments with complex backgrounds, exhibits stronger resistance to lighting and occlusion interference, and demonstrates optimal overall detection performance and practical application value in actual walnut detection tasks.

**Figure 7** shows the AP comparison chart for each category, reflecting the detection performance of the two models under four conditions: front lighting, backlighting, occlusion, and no occlusion. The AP comparison results show that the improved model outperforms the original model in all four scenarios. Specifically, the AP for front lighting increased from 76.3% to 83.9%, an improvement of 7.6 percentage points. The AP for backlighting increased from 71.7% to 81.5%, an improvement of 9.8 percentage points. The AP for occlusion increased from 66.1% to 77.6%, an improvement of 11.5 percentage points. The AP for no occlusion increased from 81.9% to 87.8%, an improvement of 5.9 percentage points. These results indicate that the improved model effectively enhances walnut detection performance, especially in complex scenarios such as backlighting and occlusion. This is because backlighting reduces the contrast between the target and the background, while occlusion causes the loss of local features in the walnut. The GhostNetV2 module strengthens global backbone modeling, the PPA module extracts information at different granularities, recapturing edges and texture changes, the DW-LAE module improves local feature extraction, and the DW Conv has learnable spatial modeling capabilities, preserving edges, textures and local details of walnut targets better during downsampling. This enhances the model’s adaptability to complex environments, reducing false positives and missed detections.

**Figure 7:**
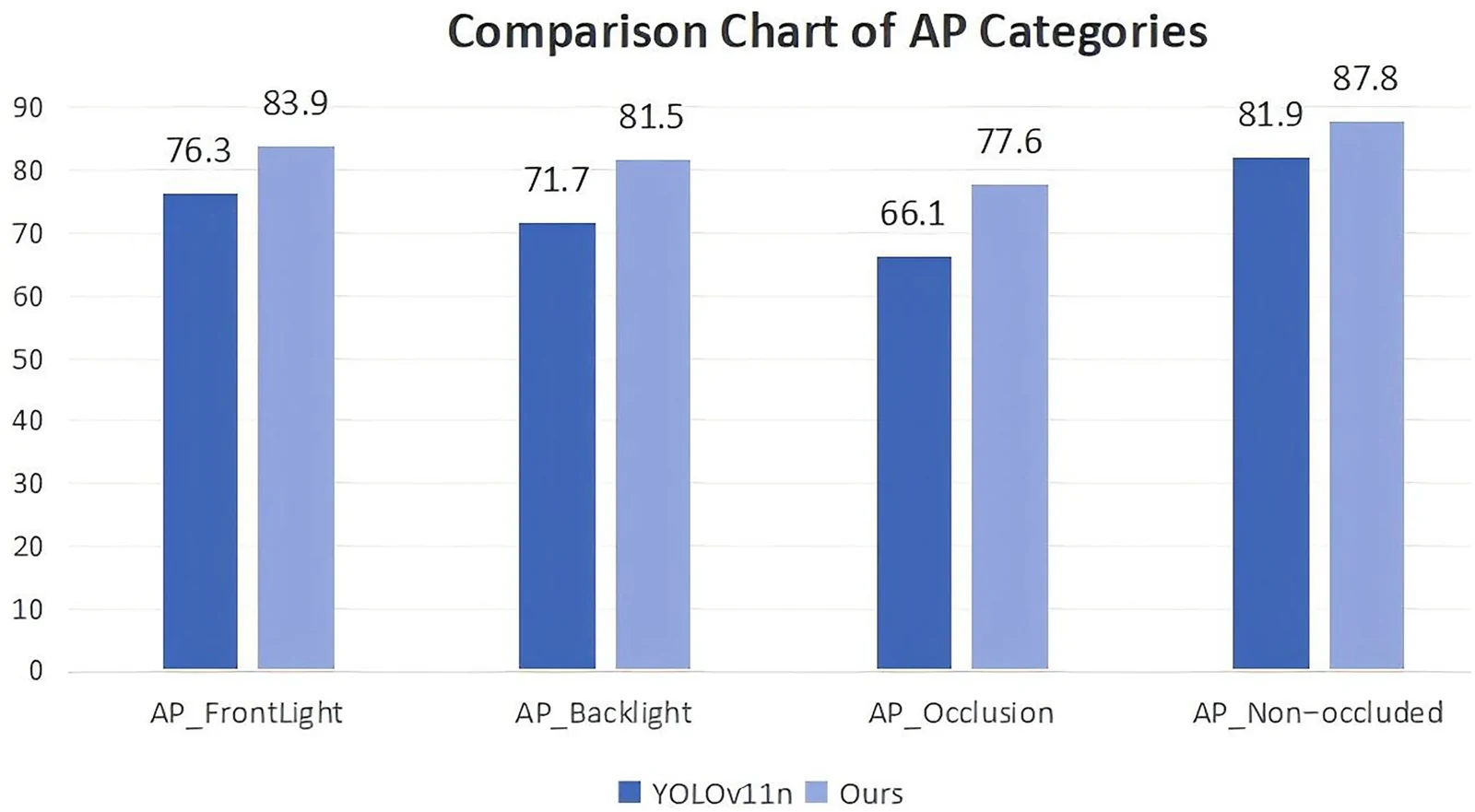
Comparison of AP by category.

**Table 2** shows the results of the DW-LAE module. DW-LAE is an average pooling operation that replaces the original LAE with deep convolution. LAE1 and LAE2 respectively represent replacing one and two convolutional layers in the YOLOv11n downsampling fusion branch with the original LAE module. LAE3 and LAE4 respectively replaced one convolutional layer and two convolutional layers with DW-LAE. The experimental results show that replacing a single convolutional layer with the original LAE module yielded only a 0.1% increase in mAP50; accuracy for the unoccluded categories A1 and A2 dropped by 0.4% and 0.3% respectively, whereas accuracy for the occluded categories B1 (front-lit) and B2 (back-lit) improved by 0.4% and 0.7%, respectively. Replacing two convolutional layers with the original LAE module resulted in no mAP50 gain; accuracy for unoccluded categories A1 and A2 decreased by 0.1%, while accuracy for occluded categories B1 and B2 improved by 0.2% and 0.3%, respectively. These findings indicate that the original LAE module mitigates the negative impact of occluded regions on overall features during the feature fusion stage; the AvgPool layer enables cross-region fusion of valid, unoccluded walnut features, thereby compensating for local feature loss in occluded walnuts and enhancing recognition precision for them. However, the original LAE module exhibits insufficient feature representation capability for unoccluded walnuts, making target features susceptible to dilution by the background; furthermore, its fixed receptive field fails to adaptively extract features from unoccluded walnuts of varying sizes, leading to reduced recognition precision for these objects. Regarding the DW-LAE module, replacing one convolutional layer resulted in a 0.1% decrease in A1 accuracy, a 0.1% increase in A2 accuracy, and improvements of 0.4% and 1% for B1 and B2, respectively. Replacing two convolutional layers with the DW-LAE module led to accuracy increases of 0.4% for A1, 0.5% for A2, 0.5% for B1, and 1% for B2. These results indicate that the DW-LAE module can maintain or enhance the recognition accuracy for occluded walnuts. Simultaneously, it can proactively learn features such as edges and surface textures of unoccluded walnuts, thereby more precisely focusing attention on the walnut region and improving the recognition accuracy for unoccluded walnuts.

**Table 6** presents the detection results of the model on the walnut dataset following the introduction of the GhostNetV2 module. GhostNetV2-1, GhostNetV2-2, GhostNetV2-3, and GhostNetV2–4 denote the replacement of one, two, three, and four C3k2 layers with GhostNetV2, respectively. The experimental results demonstrate that replacing four C3k2 layers in the backbone with GhostNetV2 yields the best performance, with accuracy improvements of 5.3% for A1, 6.4% for A2, 9.4% for B1, and 12.7% for B2. The mAP50 reached 84.4%, an increase of 8.4% over the baseline model, while mAP50:95 improved by 7.9%. These results indicate that employing the GhostNetV2 module for walnut feature extraction enhances the model’s global modeling capability, providing feature maps rich in both detailed and semantic information for the subsequent feature fusion stage.

**Table 6:** Experimental results for GhostNetV2 modules.A1, front-lit and unoccluded;. A2, back-lit and unoccluded; B1, front-lit and occluded; B2, back-lit and occluded.

Method	A1	A2	B1	B2	mAP50(%)	mAP50:95(%)	Parameters(M)	GFLOPs
Baseline(YOLOv11n)	84.0	79.7	68.5	63.7	74.0	48.0	2.6	6.3
Baseline+GhostNetV2-1	85.8	81.6	71.8	68.8	77.0	51.4	3.3	6.9
Baseline+GhostNetV2-2	86.6	82.8	73.8	71.5	78.7	52.5	4.5	7.7
Baseline+GhostNetV2-3	86.7	82.8	73.6	70.9	78.5	52.4	4.3	8.2
Baseline+GhostNetV2-4	89.3	86.1	77.9	76.4	82.4	55.9	5.1	11.0

**Table 7** presents the detection results of the model on the walnut dataset following the introduction of the PPA module. PPAs denotes the introduction of the PPA after the P3/8 multi-scale fusion feature layer, PPAm denotes its introduction after the P4/16 layer, and PPA3 denotes its introduction after the P3/8, P4/16, and P5/32 layers. Although both PPAs and PPA3 yielded improved results, PPA3 entailed a significant increase in parameter count and computational load; consequently, we ultimately chose to introduce the PPA solely after the P3/8 multi-scale fusion feature layer. The experimental results demonstrate improvements of 0.5% for A1, 0.2% for A2, 0.5% for B1, and 0.5% for B2, alongside a 0.4% increase in mAP50 and a 1.1% increase in mAP50:95. These findings indicate that the PPA module effectively extracts information at varying granularities, mitigates the loss of edge texture details caused by upsampling at the P4 layer, enhances multi-scale feature fusion capabilities, and improves localization accuracy.

**Table 7:** Experimental results for the PPA module.A1, front-lit and unoccluded;. A2, back-lit and unoccluded; B1, front-lit and occluded; B2, back-lit and occluded.

Method	A1	A2	B1	B2	mAP50(%)	mAP50:95(%)	Parameters(M)	GFLOPs
Baseline(YOLOv11n)	84.0	79.7	68.5	63.7	74.0	48.0	2.6	6.3
Baseline+PPAs	84.5	79.9	69.0	64.2	74.4	49.1	2.8	8.0
Baseline+PPAm	83.7	79.6	68.4	64.0	73.9	48.2	3.1	7.9
Baseline+PPA3	84.5	80.0	69.6	65.1	74.8	49.3	5.4	11.1

To further investigate the impact of various improvement modules on the models’ regions of feature focus and detection performance, a comparative analysis using heatmap visualization was conducted on YOLOv11n, YOLOv11n+GhostNetV2, YOLOv11n+GhostNetV2+PPA, and the final improved model, as shown in **Figure 8**. The brightness of the colors in the heatmaps reflects the intensity of the model’s activation response to different image areas; higher brightness indicates a greater degree of focus on a specific region. These visualizations allow for an intuitive assessment of how well the models focus on the target walnut regions and their ability to suppress interference from complex backgrounds.

**Figure 8:**
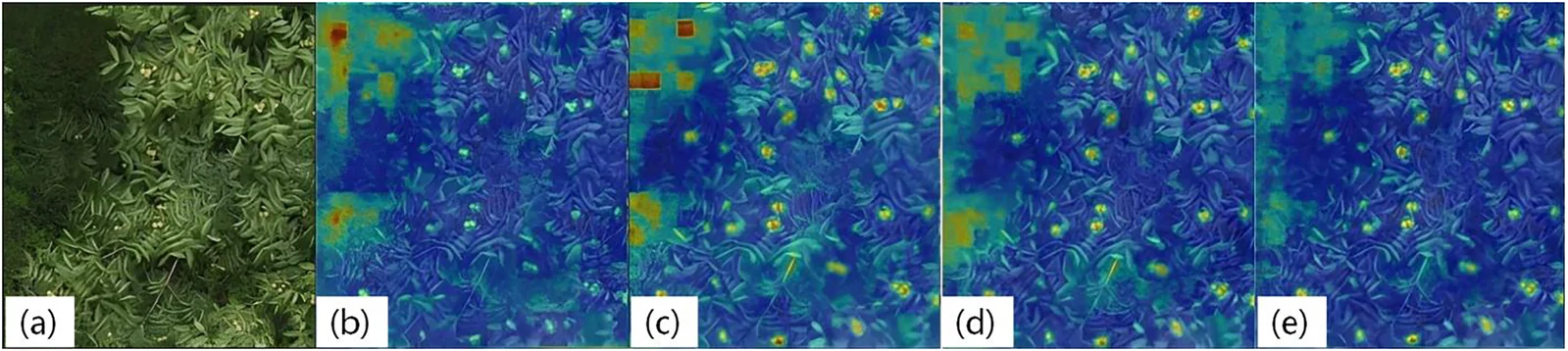
Visualization of model heatmaps under the influence of different improvement modules. **(A)** Original Image. **(B)** YOLOv11n. **(C)** YOLOv11n+GhostNetV2. **(D)** YOLOv11n+GhostNetV2+PPA. **(E)** Ours.

As shown in **Figure 8**, while the original YOLOv11n can produce some response to the walnut target, the target and background responses are similar, with some high-response areas concentrated in the background or irrelevant regions. In complex scenes, the focus on the target is not comprehensive enough. After introducing GhostNetV2, the model’s response to the walnut target region becomes more pronounced. It enhances the global modeling ability of feature extraction, supplements walnut texture and edge information, and makes the model’s focus on the target region more explicit. However, the model still suffers from high responses in local background regions, indicating that improvements to the feature extraction structure alone are insufficient to completely suppress complex background interference. Adding PPA further weakens the heatmap background response, demonstrating that the PPA module enhances the model’s ability to select key regions, allowing the network to focus more on effective information relevant to the detection task. Further adding DW-LAE results in the most concentrated heatmap response, with more pronounced responses to the walnut outline, local texture, and visible areas after occlusion, while the background response is further weakened. Especially in backlit and occluded scenes, the final model still maintains a strong target response, indicating that the DW-LAE module can retain more local detail information during downsampling and improve the model’s ability to perceive the edge and texture features of walnuts.

In summary, with the gradual introduction of GhostNetV2, PPA and DW-LAE modules, the model’s focus areas shifted from scattered to concentrated, the response of walnut target areas kept strengthening, and the response to baVckground interference gradually decreased. Heatmap visualization results show that each improved module played an active role in enhancing feature expression, highlighting key target areas, and suppressing complex background interference, further proving the effectiveness and rationality of the proposed improvements in walnut target detection in complex natural scenes.

To provide a more comprehensive evaluation of the GPL-YOLO model, the precision–recall (PR) curve and F1–confidence curve are presented in **Figures 9**, **10**, respectively. The PR curve characterizes the trade-off between precision and recall, with a larger area under the curve generally indicating better overall detection performance. As shown in **Figure 9**, the PR curve is smooth and does not exhibit any abrupt decline, suggesting that the model can maintain relatively stable precision as recall increases. This indicates that GPL-YOLO is capable of detecting a greater number of green walnut targets while limiting false-positive detections, thereby demonstrating reliable localization and recognition performance.

**Figure 9:**
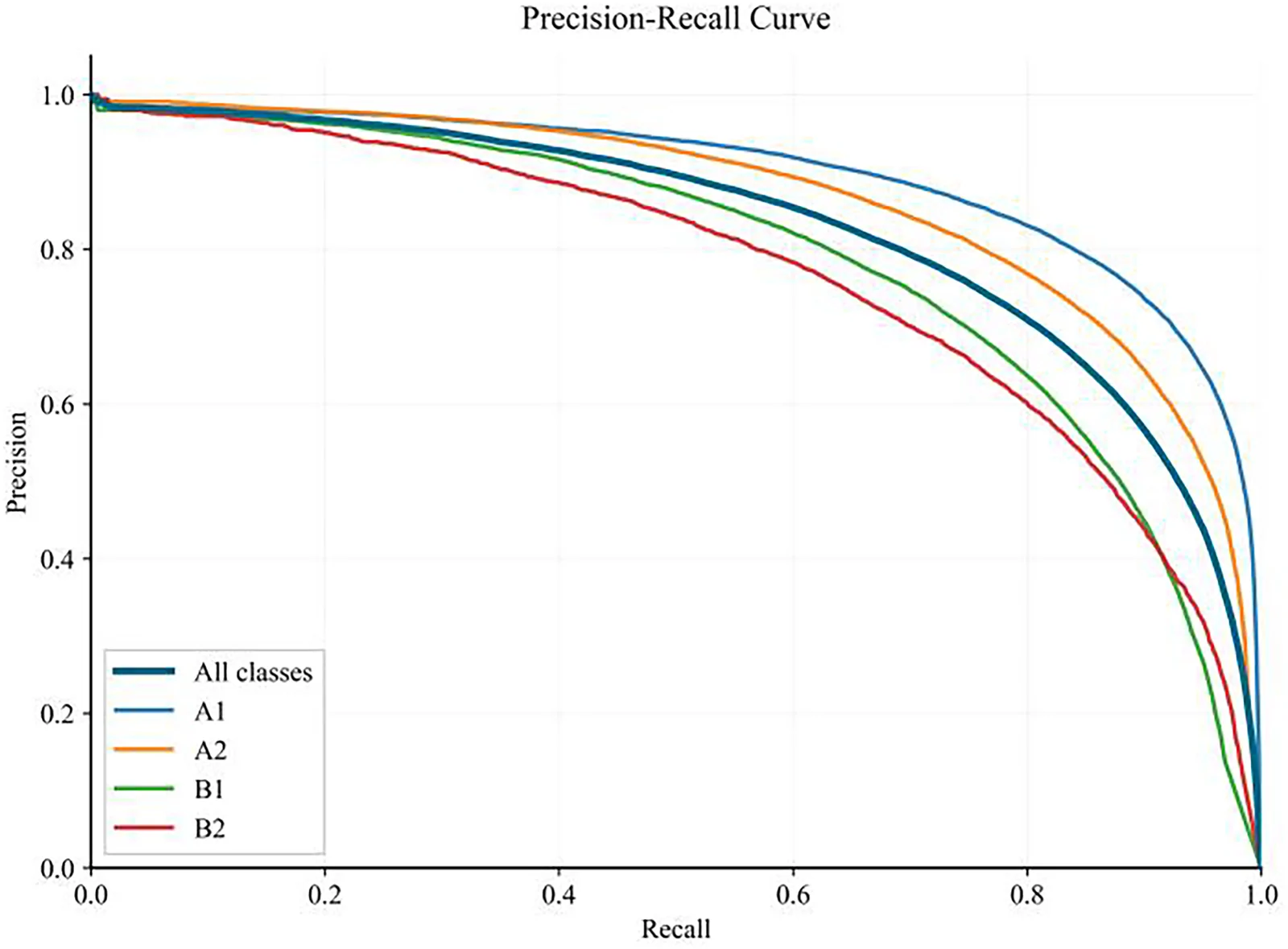
PR curve of GPL-YOLO.

**Figure 10:**
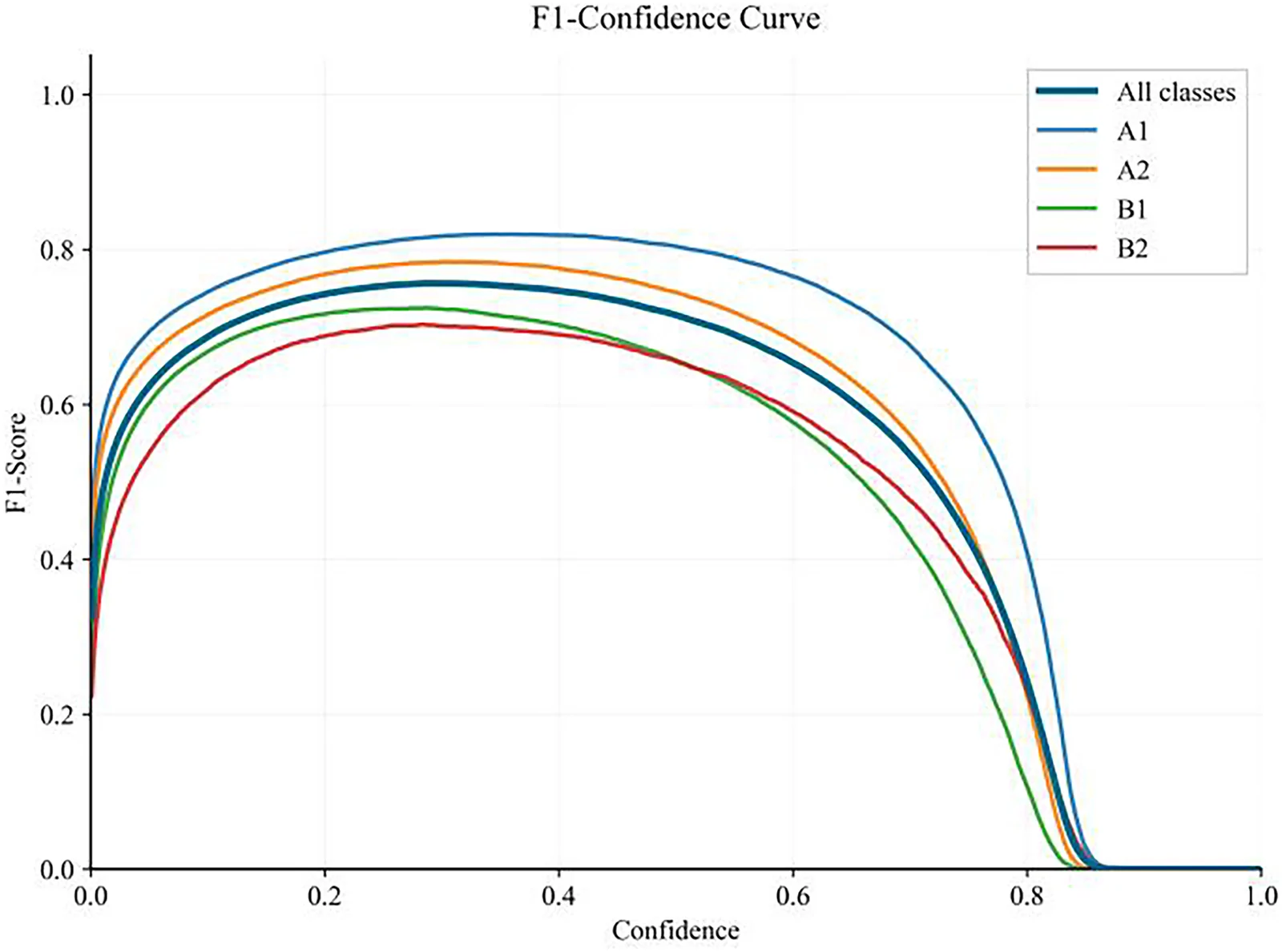
F1-Confidence curve of GPL-YOLO.

**Figure 10** shows the variation in the F1-score with the confidence threshold. As the confidence threshold increases, the F1-score first increases and then decreases, indicating that both excessively low and excessively high thresholds can compromise the overall detection performance. The maximum point of the curve represents the highest F1-score achieved at the corresponding optimal confidence threshold. The relatively gradual variation around this maximum suggests that the model is relatively insensitive to small changes in the confidence threshold and therefore exhibits good robustness for green walnut detection under complex agricultural conditions.

**Figure 11** shows the detection results on the WalnutData dataset. Yellow rectangles represent missed detections, and red rectangles represent false positives. The visualization results show that the YOLOv11n model still has a significant number of missed and false positives in walnut detection, especially when the walnuts are occluded (classes B1 and B2), where the missed and false positives are more pronounced. The figure shows that YOLOv11n easily misdetects class B1 (front-lit, occluded) walnuts as class A1 (front-lit, unoccluded) and class A2 (back-lit, unoccluded) walnuts, and easily misses class B1 walnuts. Our method can effectively reduce false positives, demonstrating the effectiveness of our method for object detection under occlusion conditions.

**Figure 11:**
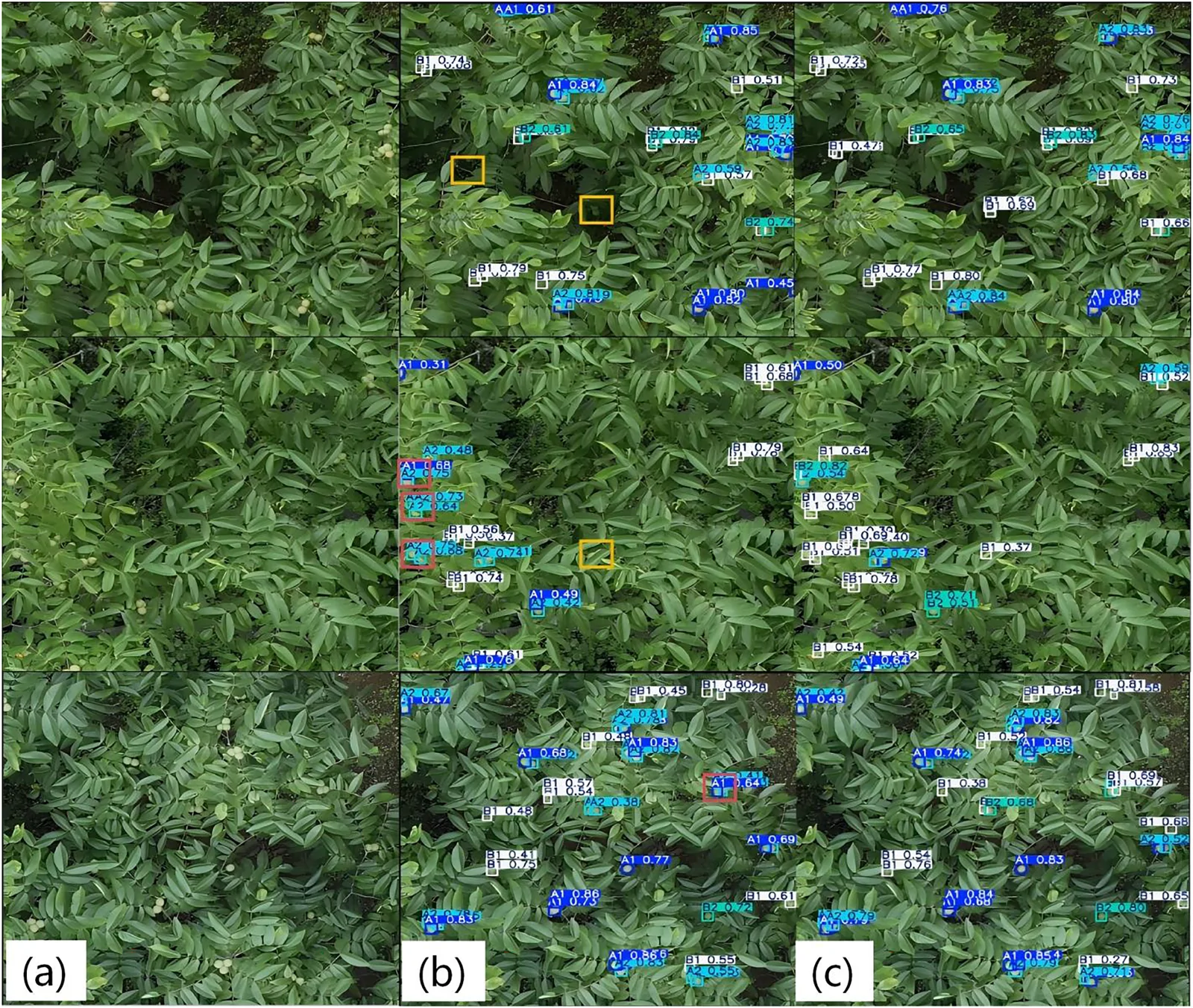
Model detection results. **(A)** Original Image. **(B)** YOLOv11n. **(C)** Ours.

## 4 Conclusions

To address the challenges of walnut detection in natural settings, such as complex backgrounds, significant lighting variations, and severe occlusion by branches and leaves, this paper proposes an improved walnut detection model based on YOLOv11n, integrating GhostNetV2, PPA, and DW-LAE modules. This model effectively enhances walnut detection performance in complex orchard environments by improving feature extraction capabilities, increasing attention to key regions, and preserving local details during downsampling.

First, this paper introduces the GhostNetV2 module into the YOLOv11n model to enhance its ability to represent walnut target features. This module can more fully extract effective information such as texture, edges, and contours of the walnut, enabling the model to have a stronger representation ability of target regions in complex backgrounds. Second, the PPA module is introduced to enhance the model’s perception of key regions, allowing the network to focus more on effective features related to walnut detection and to some extent suppressing interference from irrelevant information such as branches, shadows, and background textures. Finally, to address the problem of easy loss of local detail information of the target during downsampling, this paper proposes the DW-LAE module. This module enables the model to retain more information about the edges, texture, and local structure of the walnut during feature extraction, thereby improving the model’s detection ability in complex scenes such as occlusion, backlighting, and unclear target boundaries.

The experimental results show that the improved model achieves better detection performance for walnuts compared to the original YOLOv11n, with enhanced accuracy, recall, and mean average precision. Ablation experiments further confirm the effectiveness of the GhostNetV2, PPA, and DW-LAE modules. In summary, the improved YOLOv11n model proposed in this study can effectively boost walnut detection performance in complex natural scenes, handling challenges such as branch and leaf obstructions, backlight interference, and complex backgrounds, which can support applications like intelligent walnut recognition, orchard yield estimation, and automated harvesting.

Although the improved model proposed in this paper has achieved certain results in the walnut detection task, there are still some shortcomings that need to be further improved in subsequent studies.

First, although the dataset used in this article includes some complex natural scenes, there is still room for improvement in terms of sample size, scene diversity and coverage of extreme conditions. For example, in strong backlight, low light or rainy and foggy weather, the model’s detection stability may still be affected. Therefore, future research could further expand the walnut image dataset to improve the model’s generalization ability and robustness.

Secondly, although this paper enhances the model’s capability to extract and focus on walnut features by incorporating GhostNetV2, PPA, and DW-LAE modules, instances of missed detections or false positives may still occur in scenarios involving severe occlusion or dense distribution. Future work could further integrate more effective multi-scale feature fusion methods, contextual information modeling mechanisms, or attention mechanisms to improve the model’s recognition performance regarding small, occluded, and densely packed targets. Due to limitations in local experimental hardware computing power, this study focuses on improving detection accuracy under complex field conditions; consequently, experiments regarding model lightweighting (compression) and comparative analyses with end-to-end Transformer-based detection models (such as DETR and YOLO-Transformer) have not yet been conducted. Future work will proceed in two directions: first, optimizing the proposed model’s size and inference speed through techniques such as model pruning, quantization, and lightweight backbone networks to enable deployment on resource-constrained embedded harvesting equipment and field mobile terminals; second, expanding hardware resources to conduct multi-dimensional comparative experiments with mainstream Transformer-based models, to further validate the accuracy advantages and scenario adaptability of the proposed method for single-modality walnut detection. Overall, the improvement strategies proposed in this study effectively address the issue of insufficient walnut detection accuracy in natural orchards, offering a viable optimization approach and experimental reference for the high-precision, single-modality detection of agricultural and forestry fruits.

In addition, this article mainly focuses on walnut object detection, emphasizing the localization and identification of walnut targets, but has not yet considered practical applications such as determining walnut maturity, assessing fruit quality, or obtaining spatial location information. In the future, based on object detection models, techniques such as image segmentation, maturity recognition, depth estimation, or multi-sensor fusion could be combined to achieve a comprehensive analysis of walnut growth status, spatial position, and harvesting feasibility.

Finally, future research could further explore lightweight improvements to the model, deploying it on edge platforms to test it in real orchard environments and verify its stability and practicality. By integrating it with intelligent harvesting robots, orchard monitoring platforms, the application of intelligent walnut detection technology in smart agriculture and automated orchard management can be further promoted.

## Data Availability

The raw data supporting the conclusions of this article will be made available by the authors, without undue reservation.
